# Deep generative models for peptide design

**DOI:** 10.1039/d1dd00024a

**Published:** 2022-03-31

**Authors:** Fangping Wan, Daphne Kontogiorgos-Heintz, Cesar de la Fuente-Nunez

**Affiliations:** Machine Biology Group, Departments of Psychiatry and Microbiology, Institute for Biomedical Informatics, Institute for Translational Medicine and Therapeutics, Perelman School of Medicine, University of Pennsylvania Philadelphia Pennsylvania USA cfuente@upenn.edu; Departments of Bioengineering and Chemical and Biomolecular Engineering, School of Engineering and Applied Science, University of Pennsylvania Philadelphia Pennsylvania USA; Penn Institute for Computational Science, University of Pennsylvania Philadelphia Pennsylvania USA; Department of Computer and Information Science, School of Engineering and Applied Science, University of Pennsylvania Philadelphia Pennsylvania USA

## Abstract

Computers can already be programmed for superhuman pattern recognition of images and text. For machines to discover novel molecules, they must first be trained to sort through the many characteristics of molecules and determine which properties should be retained, suppressed, or enhanced to optimize functions of interest. Machines need to be able to understand, read, write, and eventually create new molecules. Today, this creative process relies on deep generative models, which have gained popularity since powerful deep neural networks were introduced to generative model frameworks. In recent years, they have demonstrated excellent ability to model complex distribution of real-word data (*e.g.*, images, audio, text, molecules, and biological sequences). Deep generative models can generate data beyond those provided in training samples, thus yielding an efficient and rapid tool for exploring the massive search space of high-dimensional data such as DNA/protein sequences and facilitating the design of biomolecules with desired functions. Here, we review the emerging field of deep generative models applied to peptide science. In particular, we discuss several popular deep generative model frameworks as well as their applications to generate peptides with various kinds of properties (*e.g.*, antimicrobial, anticancer, cell penetration, *etc*). We conclude our review with a discussion of current limitations and future perspectives in this emerging field.

## Abstract 2

We present a review of deep generative models and their applications in peptide design.

## Graphical abstract

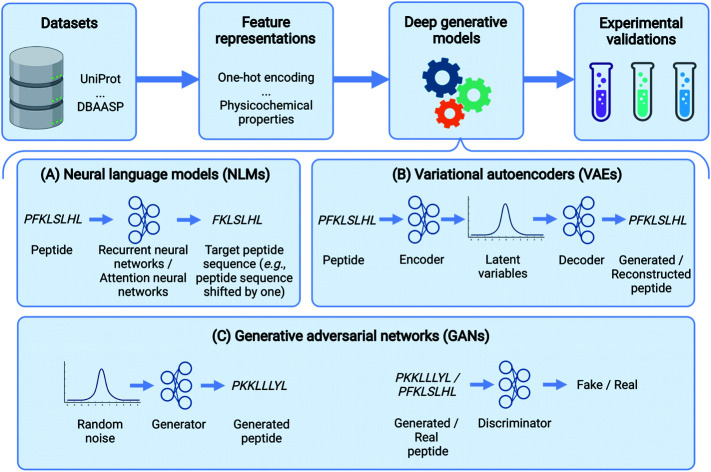

## Introduction

Defined as short chains of amino acids with lengths ranging from 2 to 50, peptides can act as hormones, antimicrobials, or delivery vehicles^1,2^ and have gained great interest due to their potential as therapeutic drugs.^1,3^ Indeed, we have witnessed an increasing number of approvals of peptide therapeutics over the past 60 years, with 52 peptide drugs approved in this century.^4^ Furthermore, over 150 and 500 peptides are in clinical trials and under pre-clinical development,^4^ respectively. Despite the potential therapeutic value of peptides,^5–7^ designing them to achieve specific properties or functions remains a challenge. The exponential search space of peptides (*e.g.*, 20^*L*^ possible peptides taking into account the 20 naturally occurring, canonical amino acids and length *L*) in addition to the high cost and time-consuming process associated with experimental validation^8–12^ pose serious issues when developing peptide drugs.

In recent years, a data-driven paradigm has emerged by combining deep learning and advanced computational resources [*i.e.*, graphics processing units (GPUs)], revolutionizing a number of fields, including computer vision,^13^ natural language processing (NLP),^14^ game playing,^15^ and computational biology.^16–19^ As universal approximators,^20^ deep neural networks have demonstrated superior abilities in modelling complex real-world data, including extracting high-level features from raw inputs,^21,22^ making predictions,^13^ fitting data distributions, and generating novel data.^23–25^ Among various kinds of deep learning frameworks, deep generative models differ from classification and regression models (*i.e.*, discriminative models) in their ability to model the distribution of data using deep neural networks. Consequently, properly trained deep generative models can be used to (1) assign a likelihood/probability to measure if a novel data point is drawn from the data distribution, (2) sample and/or generate novel data points that possess similar properties to those present in the training data, and (3) extract expressive data representations^23,24^ (*i.e.*, feature learning) or perform casual inference^26^ (*e.g.*, determine the casual factor that lead to the generation of data) by specifying the generation process of data. In a number of application fields, deep generative models have exhibited superior performance in generating complicated and high-dimensional data, including realistic images,^27,28^ syntactically correct programming language codes,^29^ and drug-like molecules.^30^ Deep generative models could potentially serve as a new tool to efficiently explore the vast peptide sequence space and facilitate the peptide design process by prioritizing promising peptides for experimental validation.

**Fig. 1:**
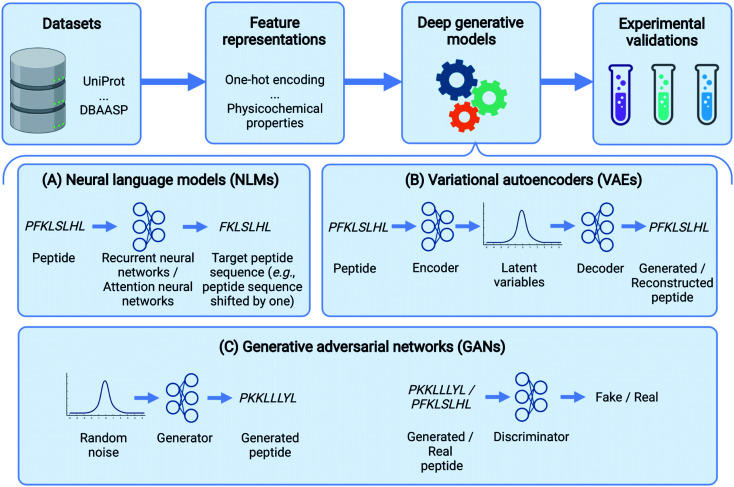
Peptide design pipeline based on deep generative models. This data-driven approach starts with peptide data curation and conversion of peptides to machine-readable representations. Deep generative models generate novel peptides by taking the above representations and modeling the distribution of the training peptide data. The generated peptides are then examined and validated by wet-lab experimentation. Among various deep generative models, Neural language models (NLMs) either predict the next amino acid based on previously generated amino acids or map the source peptides to target ones. Variational autoencoders (VAEs) use an encoder and a decoder to map peptides to latent variables and generate peptides from latent variables, respectively. In generative adversarial networks (GANs), a generator produces synthetic data while a discriminator distinguishes generated samples from real ones.

In the past few years, deep learning methods have also been widely used in peptide science to perform various tasks like peptide identification, property prediction, and peptide generation.^31^ Specifically, for peptide generation, deep generative models have been used to generate peptides with a range of activities, including the following: antimicrobial, anticancer, immunogenic, ability to deliver other molecules, and signal peptides (Table 1). Here, we comprehensively review these deep generative models for peptide generation. Several reviews have described the use of deep generative models for proteins.^32,33^ Our paper, instead, focuses exclusively on peptide design. We should also point out that the focus of deep generative models on peptide design inevitably leads to a neglect of some generative models including flow-based^34^ and energy-based models.^35^ We refer the reader to a recent review^33^ for further details. Specifically, the rest of the paper is organized as follows: Before delving into specific generative algorithms, we first describe several commonly used datasets and feature representations of peptides. We then provide an overview of three popular deep generative model frameworks used in peptide generation: neural language models (NLMs), variational autoencoders (VAEs), and generative adversarial networks (GANs) (Fig. 1). The basic concepts of these models, as well as those of their variants, will be introduced and their applications in peptide generation will be reviewed. Finally, we conclude by discussing outstanding challenges and future directions in this exciting field. Although this review is primarily aimed at readers with a basic understanding of machine learning and deep neural networks, our goal is to reach a broader readership. Therefore, a glossary is provided (Table 2) to cover definitions of key machine learning terms that are not strictly defined in main text.

**Table 1:** Peptide generation studies using deep generative models. Abbreviations: NML, neural language model; VAE, variational autoencoder; GAN, generative adversarial network; AMP, antimicrobial peptide; ACP, anticancer peptide; CPP, cell-penetrating peptide; PMO, phosphorodiamidate morpholino oligomer

Method	Feature Representation	Application	Citation	Year
NML	One-hot	AMP generation	Müller *et al.*^56^	2018
NLM	Character sequence	AMP generation	Nagarajan *et al.*^52^	2018
NLM	Character sequence	ACP generation	Grisoni *et al.*^46^	2018
NLM	Learned representation using one-hot	Signal peptide generation	Wu *et al.*^55^	2020
NLM	Learned representation using structural and evolutionary data	AMP generation	Caceres-Delpiano *et al.*^54^	2020
NLM	One-hot	AMP generation	Wang *et al.*^41^	2021
NLM	Character sequence	CPP generation	Tran *et al.*^53^	2021
NLM	One-hot	AMP generation	Capecchi *et al.*^42^	2021
NLM	Fingerprint, one-hot	PMO delivery peptide generation	Schissel *et al.*^57^	2021
VAE	Learned representation using character sequence	AMP generation	Das *et al.*^38^	2018
VAE	Learned representation using one-hot	AMP generation	Dean *et al.*^44^	2020
VAE	Learned representation using character sequence	AMP generation	Das *et al.*^45^	2021
GAN	Character sequence	AMP generation	Tucs *et al.*^50^	2020
GAN	Character sequence/PDB structure	ACP generation	Rossetto *et al.*^48^	2020
GAN	Learned representation using character sequence	AMP generation	Ferrell *et al.*^39^	2020
GAN	Character sequence	AMP generation	Oort *et al.*^40^	2021
GAN	Sequence of amino acid property vectors	Immunogenic peptide generation	Li *et al.*^51^	2021
GAN	Character sequence	AMP generation	Surana *et al.*^49^	2021

**Table 2:** Glossary of machine learning terms that are not strictly defined in this review. All terms are delineated in alphabetical order

Term	Meaning
Active learning	Algorithms to query data samples from a dataset for labelling and model training so that the trained model has maximum performance gain
Backpropagation	An algorithm that trains neural networks. Gradients of the loss with respect to parameters in a neural net are first calculated by the chain rule. Then, gradient descent is performed to optimize the parameters
Bayesian optimization	A sequential search algorithm to optimize computationally expensive black-box functions
Bidirectional encoder representations from transformers	Transformer-based language model for feature/representation learning
Convolutional neural network	A class of neural networks that uses a series of convolution operations and nonlinear transformations to process structured inputs (*e.g.*, images and sequences) and make predictions
Data augmentation	Supplementing a dataset with modified copies of the data or with synthetic data, often used to prevent overfitting and improve prediction performance
Decoder	A neural network converts compressed signals/features (usually represented as low-dimensional vectors) to raw signals
Embedding	A mapping from a high-dimensional input to a low-dimensional vector
Encoder	A neural network converts raw inputs/signals to compressed signals/features (usually represented as low-dimensional vectors)
Gradient	In machine learning, it usually refers to the partial derivative of loss with respect to machine learning model (*e.g.*, neural network) parameters
Gradient descent	An optimization algorithm to minimize a differentiable function by iteratively moving in the opposite direction of the gradient of the function
Label	The answer of what machine learning models aim to predict
Loss	A measure between label and machine learning model prediction, indicating how far the prediction is from the corresponding label
Low-shot learning	Machine learning approaches that train effective models using a small number of training samples. Also known as few-shot learning
Multitask learning	Machine learning approaches that train models to solve multiple tasks simultaneously
Neural network	A model inspired by the brain that uses a series of nonlinear transformations to process inputs and make predictions
Objective	A value (*e.g.*, loss) that machine learning models aim to minimize/maximize
One-hot encoding	A vector representation of categorical data, wherein all values are 0 except the singular 1
Recurrent neural network	A class of neural networks that are suitable for modelling time series and sequential data by using the output from the last time step as input to the current time step
Training	A process to optimize parameters of machine learning models so that objective(s) are minimized/maximized
Training data/dataset/set	Data used to train machine learning models
Transfer learning	Machine learning approaches that transfer information/knowledge from one task to the other in order to improve prediction performance of models
Transformer	A neural network architecture based on attention for sequence-to-sequence learning

### Datasets

While there are publicly available datasets for protein informatics with labeled protein activity, their size is limited, particularly compared with the extensive datasets available in NLP and computer vision. The scarcity of data in this case is primarily due to the cost associated with running wet-lab experiments. For example, WordNet has over 150 000 well-organized words, and ImageNet has over 14 million labeled images for object recognition.^36,37^ On the contrary, as shown in Table 3, the size of databases with labeled data used by many of the studies covered in this review are in the order of hundreds or thousands. Further, the actual size of the datasets used in these studies is usually much smaller, after filtering for how activity was measured, specific targets, peptide length, presence or absence of non-canonical residues, unique sequences, synthesis difficulty, and other properties that help build a high-quality dataset. For properties such as antimicrobial activity, labeled negative data are often even more scarce than positive. To address the negative data scarcity problem and improve the generalizability of these models, many studies^39–42^ have resorted to using unlabeled proteins from databases like UniProt^43^ and DBAASP^73^ or randomly generated sequences^42^ as negative examples. The validity of this method is supported by Wang *et al.*^41^ To best address this issue, however, the field relies on experimentalists collecting and sharing data on peptides negative data on peptides. As an alternative to assuming unlabeled data is negative, unsupervised learning can take advantage of unlabeled data to learn feature representations. For example, Das *et al.*^45^ trained a general peptide generation model based on peptides from UniProt, whereas Grisoni *et al.*^46^ and Capecchi *et al.*^42^ incorporated transfer learning to be able to utilize more data.

**Table 3:** Peptide databases covered in this review

Name	Citation	Labels	Data size	Application
Uniprot	The UniProt Consortium^43^	Sparse labels	190 million sequences	Wu *et al.*^55^
				Das *et al.*^38^
				Capecchi *et al.*^42^
				Das *et al.*^45^
				Oort *et al.*^40^
CPPsite 2.0	Agrawal *et al.*^116^	Cell-penetrating peptides	1850 peptides,1,150 used in application	Schissel *et al.*^57^
Pfam	Bateman *et al.*^117^	Sparse labels	47 million sequences, 21 million used in application	Caceres-Delpiano *et al.*^54^
DBAASP	Pirtskhalava *et al.*^73^	Antimicrobial, toxicity, anticancer and hemolytic activity	>15, 700 peptides	Wang *et al.*^41^
				Tran *et al.*^53^
				Capecchi *et al.*^42^
				Das *et al.*^45^
				Tucs *et al.*^50^
				Ferrell *et al.*^39^
				Oort *et al.*^40^
ToxinPred's dataset	Gupta *et al.*^118^	Toxicity	1805 toxic peptides	Das *et al.*^45^
AVPdb	Qureshi *et al.*^119^	Antiviral	2683 peptides	Oort *et al.*,^40^
				Surana *et al.*^49^
LAMP	Zhao *et al.*^120^	Antimicrobial activity	5547 peptides	Tran *et al.*^53^
				Tucs *et al.*^50^
THPdb	Usmani *et al.*^121^	FDA approved therapeutic peptides	239 peptides	Rossetto *et al.*^48^
CAMP	Thomas *et al.*^122^	Antimicrobial activity	3782 peptides	Wang *et al.*^41^
				Tran *et al.*^53^
				Tucs *et al.*^50^
				Surana *et al.*^49^
DRAMP	Kang *et al.*^123^	Antimicrobial activity	19 899 peptides	Wang *et al.*^41^
				Surana *et al.*^49^
YADAMP	Piotto *et al.*^124^	Antimicrobial activity	2133 peptides	Nagarajan *et al.*^52^
				Wang *et al.*^41^
DADP	Novkovi'c *et al.*^125^	Broad defence activity	2571 peptides	Müller *et al.*^56^
ADP	Wang *et al.*^126^	Antimicrobial activity	3273 peptides	Müller *et al.*^56^
				Tran *et al.*^53^
				Dean *et al.*^44^
				Tucs *et al.*^50^
DBAMP	Jhong *et al.*^127^	Antimicrobial activity	12 389 peptides	Surana *et al.*^49^
IEDB	Fleri *et al.*^128^	Immune epitope	> 1 million peptides, 8971 used in application	Li *et al.*^51^

### Feature representations

A critical component of any machine learning task is the feature selection and input representation. An extra constraint imposed on the representation schemes used in peptide generative tasks, which does not apply broadly to all peptide property prediction tasks, is the need for a mapping of the input representation back to the peptide sequence. For example, global properties and amino acid composition have been extensively used to represent peptides in property prediction tasks,^47^ but an inverse mapping of a peptide represented by global properties back to a sequence currently does not exist. However, some peptide generation studies have used representations that cannot be mapped directly to a unique sequence in property predictors to filter out those peptides generated that possess undesired properties. For instance, Rossetto *et al.*^48^ used a 4D tensor representing structural information to calculate a reward function for the generated peptide, and Surana *et al.*^49^ used physicochemical properties, amino acid composition, and structural information to analyze the generated peptides. Schemes commonly used to represent peptides in the generation task itself include direct sequence representation^41,42,45,46,49–53^ and learned embeddings.^38,39,44,45,54,55^

A natural way to encode peptides is through their primary structure (*i.e.*, amino acid sequence). A peptide of length *L* can be represented by a string of characters or integers of length *L*,^39,45,46,49,50^ or a *L* × *n* matrix such that each amino acid has a unique *n*-dimensional vector. The *n*-dimensional vector may represent either experimentally^51^ or computationally derived properties,^45^ or be a one-hot encoding.^41,42,44,56^ In a one-hot encoding, the *i*^th^ amino acid from an alphabet of size *n* is represented by a vector containing *n* − 1 0s and a 1 at the index *i*. Some studies use amino acid alphabets that expand beyond the standard 20 amino acids to represent non-canonical amino acids, markers for modified peptide terminals (*e.g.*, an acetylated N-terminal), and padding (to allow encoding peptides of different lengths). A one-hot encoding does not retain information about amino acids, such as the similarity between leucine and isoleucine; however, gains in model performance when using more complex representations may be limited.^51^ Schissel *et al.*^57^ represented amino acids as topological fingerprints. These fingerprints contain bits to represent the presence of substructures and hence retain structural information about each residue. The authors found that such an encoding scheme led to models with lower accuracies, but with an enhanced generalizability to peptides with labels outside the range of the training data. ElAbd *et al.*^58^ showed that deep learning models can learn the amino representation scheme, whether it is a one-hot encoding or even a random vector, provided that the random vector has sufficiently high dimensionality. A substantial flaw in one-hot encodings, however, is their high dimensionality.^58^ Instead of using single amino acid residues as the smallest unit within a sequence, a k-mer can be considered the fundamental unit. A *k*-mer is a short amino acid sequence of length *k*, such as a 3-mer. These *k*-mers can be represented with a one-hot encoding. Depending on the choice of *k*, the dimensionality of this representation can explode, because there are many possible *k*-length amino acid combinations. However, using an embedding can reduce the dimensionality by orders of magnitude and ameliorate this problem.^59^

In addition to sequence representation, studies have focused on learning a latent representation from a deep neural network, such as an autoencoder.^38,44,45^ This latent representation can be used solely for the purpose of sequence generation,^44^ or additionally in property prediction models.^45^ These studies all used variational autoencoders, which can learn both a feature encoding and a distribution of the inputs to then generate peptides retaining properties present within the training set. However, a classical autoencoder (*i.e.*, deterministic autoencoder) – which learns only feature encoding and its mapping to and from the sequence – could be used for the purpose of representation learning alone. Further, latent spaces leveraged to represent peptides can be learned from other model architectures, such as a GAN^60^ or an attention network like Bidirectional Encoder Representations from Transformers (BERT).^22^

### Deep generative models

In this section, we delineate the basic concepts underlying several deep learning-based generative models (Fig. 1) and summarize their applications for peptide generation (Table 1).

### Neural language models (NLMs)

In NLP, given a sequence of words **x** = (*w*_1_, *w*_2_, …, *w*_*n*_), language models estimate the probability distribution 
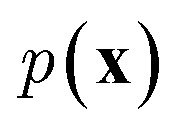
 over it. How 
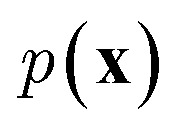
 is factorized can reflect the generation process of a sentence. Here, a popular choice (but not the only one) to specify the generation process of a word sequence is based on the chain rule of probability:1
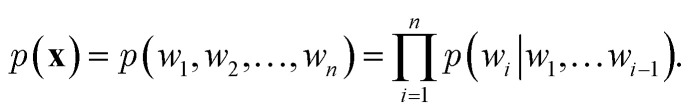


The models adopted in the generation process of eqn (1) are also known autoregressive models. That is, the next word *w*_*i*_ is generated by taking all its previous words into account. In the context of deep learning, NLMs utilize neural networks to model the conditional probabilities of eqn (1). Here, we introduce two frameworks that are widely used in NLMs: recurrent neural networks (RNNs)^61^ and attention models.^14,62^ Note that it is also possible to use other network architecture like convolutional neural network as an autoregressive model.^63^

Specifically, RNNs such as long short-term memory (LSTMs)^64^ and gated recurrent unit (GRU)^65^ are commonly used to build autoregressive models as they model sequential data by storing the historical information (*i.e.*, memory) into their hidden states. In the context of language modeling, RNNs are typically trained to predict the next word *w*_*i*+1_ given the current input word *w*_*i*_ and the hidden state **h**_*i*−1_ that stores the information from word *w*_1_ to *w*_*i*−1_:2
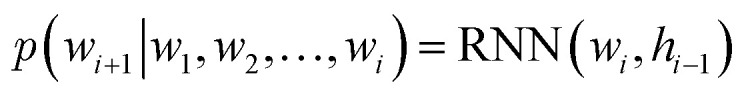
which is equivalent to maximizing the marginal likelihood 
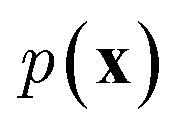
 of a sequence of words in the training data. To generate novel sequences, a desired/random start word as well as a zero/random hidden state are first provided to RNNs to generate the distribution of the next word. Then, the same process is repeated by sampling a word from the predicted distribution and inputting it to the RNNs until some termination criterion is met (*e.g.*, an “end” word is generated or a maximum generation length is reached).

While RNNs implicitly model the long-range interactions of inputs by assuming the hidden states always retain all previous input information (eqn (2)), attention models utilize neural networks to directly model such interactions. Generally, given a feature sequence (**x**_1_, **x**_2_, …, **x**_*n*_), an attention model can be formulated as:3
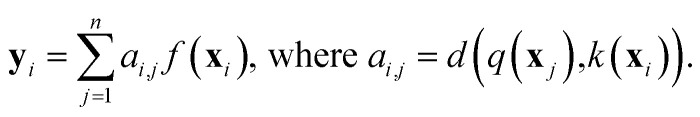
Here *f*(·), *q*(·) and *k*(·) are neural networks for nonlinear feature transformations, *d*(·,·) is a neural network to model the pairwise feature interaction given a pair (*q*(**x**_*j*_), *k*(**x**_*i*_)) and generate a scalar value to represent its interaction strength, and **y**_*i*_ is the output of the attention model with respect to input **x**_*i*_. Note that *a*_*i*,*j*_ is usually post-processed by a softmax function to ensure 
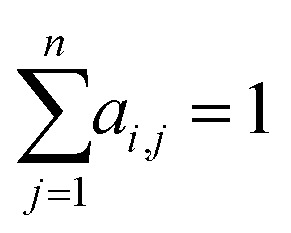
 or treated as a parameter to sample a binary value (*i.e.*, 0 or 1)^66^ as its replacement in eqn (3) (also known as hard attention). Unlike RNNs, attention models do not utilize the sequential information of input data. To address this issue, positional information like positional embedding^14^ can be incorporated into input features. To model the generation of a sequence, eqn (3) can be modified as:4
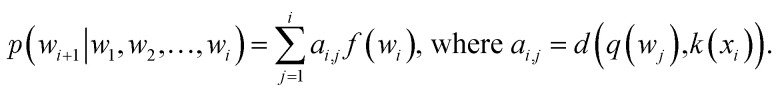
Here, the future word information (*i.e.*, *w*_*i*+2_, *w*_*i*+3_, …, *w*_*n*_) is removed from the attention model to prevent information leakage. In recent state-of-the-art language feature learning model BERT,^22^ the loss for model training eqn (4) is replaced by a mask loss (*i.e.*, attention network is required to predict words that are masked from input based from context) and a “next-sentence” loss (*i.e.*, predict the probability of next sentence given previous sentences). Currently, attention models have been widely adopted to model proteins.^67–70^

The above NLMs can be further extended to perform sequence to sequence (seq2seq) modeling,^71^ in which we aim to learn a mapping from a source sequence (*x*_1_, *x*_2_, …, *x*_*m*_) to a target sequence (*y*_1_, *y*_2_, …, *y*_*n*_). For instance, Winter *et al.*^72^ trained a RNN-based seq2seq method to obtain expressive latent features for compounds by translating the compounds' IUPAC representations to SMILES representations.

For peptide design and discovery, by treating peptides as sequences of amino acids, NLMs have been widely used to generate antimicrobial,^41,42,52,54,56^ anticancer,^46^ cell-penetrating,^53^ phosphorodiamidate morpholino oligomer (PMO) delivery,^57^ and signal^55^ peptides. To generate antimicrobial peptides (AMPs), previous work^41,42,52,56^ focused on using RNNs to model AMPs acquired from public datasets. Then, property filters like AMP predictors (*i.e.*, trained classifiers or public prediction servers) were used to remove undesired peptide sequences generated by RNNs. In addition to these approaches, Capecchi *et al.*^42^ utilized a transfer learning strategy to fine tune the RNN model trained on DBAASP^73^ to two smaller non-hemolytic AMP datasets in order to address the scarcity of non-hemolytic AMP data. Taking a different approach, Caceres-Delpiano *et al.*^54^ adopted a multitask training strategy to simultaneously perform language modeling, secondary structure, contact map, and structure similarity prediction. After model training, novel peptide sequences were generated by mutating the target sequence and after meeting certain structure similarity and energy criteria based on the trained multitask model. Through wet-lab validation, Nagarajan,^52^ Capecchi *et al.*,^42^ and Caceres-Delpiano *et al.*^54^ managed to identify two, eight, and two novel AMPs, respectively. Grisoni^46^ also used transfer learning to fine tune the RNNs trained on 10K *α*-helical cationic amphipathic peptide sequences to 26 known anticancer peptides (ACPs). The authors further experimentally validated the anticancer activity of the ten generated peptides. Although both leveraged RNNs to generate cell-penetrating peptides (CPPs), Tran *et al.*^53^ used molecular dynamics simulations to prioritize generated CPPs for downstream validation, whereas Schissel *et al.*^57^ further utilized a deep learning-based PMO delivery predictor as well as a genetic algorithm to optimize the generated CPPs to PMO delivery peptides and demonstrated their safety and efficacy in animals. Moreover, Wu *et al.*^55^ formulated the signal peptide (SP) generation challenge as a machine translation problem (*i.e.*, seq2seq), in which the transformer^14^ model was used to translate a mature protein with the SP sequence removed to the corresponding SP sequence. Furthermore, the authors validated the generated SPs by demonstrating their industrial-level enzyme secretion activity.

### Variational autoencoders (VAEs)

VAEs are similar to classic autoencoders in terms of their model architecture, which typically consist of an encoder and a decoder neural network. However, they differ substantially in mathematical formulation. Merging probabilistic graphical models and deep learning, VAEs^23,24^ assume the data is generated from certain latent variables and provide a systematic approach to model data generation (*i.e.*, decoding process) and infer the latent variables (*i.e.*, encoding process) based on deep neural networks. On the one hand, the decoder neural network *d*(·) explicitly specifies how the observed data **x** can be generated given the latent variables **z** (*i.e.*, **x** = *d*(**z**)) as well as the corresponding likelihood 
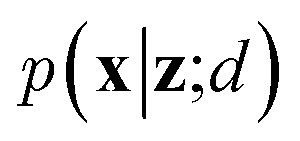
. On the other hand, given the observed data **x**, the encoder neural network *e*(·) is responsible for estimating posterior distribution 
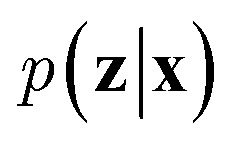
*via* variational posterior *q*(**z**|**x**;*e*), where **z** = *e*(**x**). Here, the introduction of low-dimensional latent variables subjected to certain prior distribution 
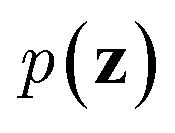
 not only makes it easier to model high-dimensional data (*e.g.*, images, text and biological sequences), but also allows users to draw samples 
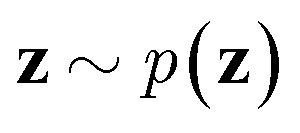
 from the prior distribution and obtain novel data through decoding.

To train VAEs, we want to maximize the marginal likelihood with respect to the parameters of *e*(·) and *d*(·):5
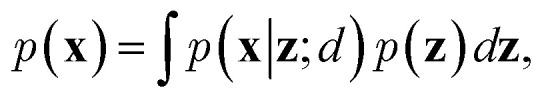
which is intractable. Therefore, the original VAEs^23^ seek to maximize a lower bound of eqn (5) (*i.e.*, evidence lower bound):6
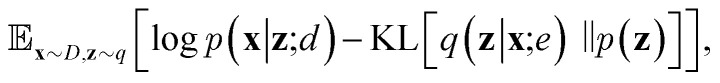
where *D* is the training dataset and KL[·‖·] is the Kullback-Leibler (KL) divergence. The first term of eqn (6) measures the reconstruction quality of VAEs whereas the second term matches the inferred latent variables to prior distribution (*e.g.*, multivariate normal distribution). In practice, we sample mini-batches from the training dataset and jointly optimize the encoder and decoder networks through gradient descent *via* backpropagation. It should be noted that directly sampling **z** from *q*(·) is a non-continuous operation and the gradients cannot be propagated to the encoder. A reparameterization trick is needed to address this issue. We refer the reader to the original paper^23^ for further details.

Since the introduction of VAEs, a number of variants were proposed to further enhance their modeling abilities. For instance, hierarchical VAEs introduced multiple layers of latent variables to create richer prior distributions.^74,75^ To incorporate supervised information into a VAE framework, conditional VAEs (CVAEs)^76,77^ introduced label variable **y** into the processes of data generation 
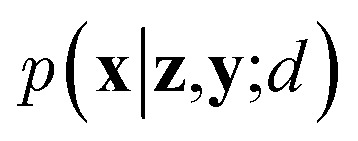
 and posterior inference *q*(**z**|**x**,**y**;*e*). Instead of randomly sampling the latent variables from prior distribution, CVAEs explicitly specify the label variable during decoding and generate samples (*i.e.*, conditional sampling) with the desired properties.^78^ Moreover, methods like maximum mean discrepancy VAE (MMD-VAE)^79^ and β-VAE^80^ replaced the original KL divergence term in eqn (6) with alternatives to encourage the models to learn more meaningful latent variables (*i.e.*, features). Today, VAEs have been widely used in various types of tasks in computer vision,^81,82^ NLP,^78,83^ recommendation systems,^84^ and bioinformatics.^17,30,85^

In the context of peptide generation, VAEs have mainly been used to discover AMPs.^38,44,45^ Dean *et al.*^44^ directly trained a VAE to model 6K peptides derived from an AMP database. Das *et al.*,^38,45^ on the other hand, utilized a larger unlabeled peptide dataset obtained from Uniprot to train the VAEs. Specifically, PepCVAE^38^ is a semi-supervised learning framework based on CVAE to jointly model 15K labeled AMPs and non-AMPs as well as 1.7M unlabeled peptide sequences from UniProt. By explicitly disentangling the AMP/non-AMP property from other latent features, PepCVAE was able to control the generation of peptides with desired AMP/non-AMP properties. Although trained on similar data, the VAE framework described in Das *et al.*^45^ improved the peptide generation process by replacing KL divergence loss with MMD loss to get more meaningful latent representations of peptides. To sample peptides with desired properties (*e.g.*, AMPs and low toxicity), the authors first fitted a Gaussian mixture density estimator and linear property predictors on latent variables of labeled peptide data. Then, they developed a rejection sampling to sample desired latent variables from the density estimator with probability derived from the property predictors and obtained peptides by passing the sampled latent variables to the decoder of the VAE. Instead of sampling from prior distribution, the authors argued that the learnt distribution of latent variables *q*(**z**|**x**;*d*) could be quite different from the prior distribution and therefore a separate density estimator was required. Das *et al.* also showed that the combination of their VAE framework with molecular dynamics simulations and wet-lab experimentation yielded two novel and safe AMPs within 48 days. These studies demonstrate the potential of VAEs in peptide drug discovery and development.

### Generative adversarial networks (GANs)

Inspired by game theory, GANs^25^ assume a two-player zero-sum game scenario, in which a discriminator *d* aims to distinguish fake and real data while a generator *g* tries to generate fake data as realistic as possible in order to fool the discriminator. As this competition between generator *g* and discriminator *d* proceeds, an equilibrium occurs where the fake examples generated by *g* are indistinguishable from real ones and *d* can only take a random guess as to whether a given example is real or not. In such an equilibrium, no further improvement can be made for *g* and *d*, and consequently we consider that the generator *g* captures the distribution of real data. In practice, both generator *g* and discriminator *d* are usually implemented as deep neural networks. For generator neural network *g*(·), it typically takes a random vector **z** drawn from a noise distribution 
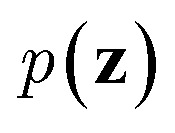
 (*e.g.*, standard Gaussian distribution) as input and outputs the generated data **x**_fake_ = *d*(**z**). For classic discriminator neural network *d*(·)→(0,1) (*i.e.*, a classifier), it receives both real samples **x**_real_ drawn from training dataset 
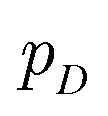
 as well as fake samples **x**_fake_ and classifies them to be real (*i.e.*, *d*(**x**_real_)→1) or not (*i.e.*, *d*(**x**_fake_)→0), respectively. The above competition process between generator and discriminator can be mathematically formulated as a minimax loss function to optimize the learnable parameters of *g*(·) and *d*(·):7
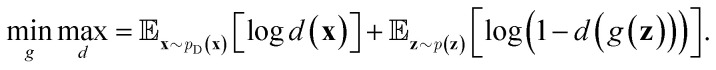


While the previously described NLMs and VAEs are explicit density models that specify the form of 
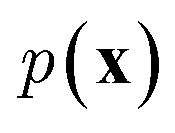
 and optimize the models to maximize 
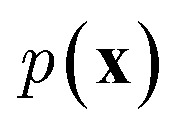
 or its lower bound, GANs use implicit density models. From the model architecture and loss function shown in eqn (7), we can see that GANs do not fit and estimate the data distribution 
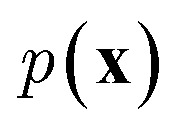
 directly. However, the original GANs paper^25^ showed that training GANs implicitly minimized the Jensen-Shannon divergence (JSD) between generated data distribution 
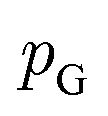
 and real data distribution 
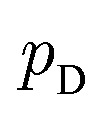
. Similar to VAEs, GANs can be extended to incorporate supervised information. By enabling generator **x** = *g*(**z**,**y**) and discriminator *d*(**x**,**y**)→(0,1) to take label information **y** into account, conditional GANs (CGANs)^86,87^ demonstrated their ability to generate data possessing desired properties. Without modifying the distribution of training data, CGANs and CVAEs encourages the models to draw samples with desired properties by introducing label information during sampling. FB-GAN,^88^ on the other hand, encourages the models to capture the sample distribution with desired properties by shifting the distribution of training data during model training. Specifically, an external property predictor is introduced to iteratively replace the data point with predicted undesired property with generated data point with predicted desired property.

Although GANs have received great attention since they came out in 2014, problems like gradients vanishing^89^ and difficulty in converging to equilibrium^90^ make GANs notoriously difficult and unstable to train. To address these issues, a number of loss functions were proposed to replace the JSD.^91–96^ Among these variants, we discuss a widely adopted framework called Wasserstein GANs (WGANs).^95^ In WGANs, instead of being a binary classifier, the discriminator *d*(·) is used to measure the Wasserstein distance. Specifically, the Wasserstein distance (also known as earth mover's distance) of two distributions 
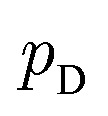
 and 
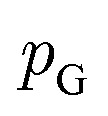
 can be mathematically written as:8
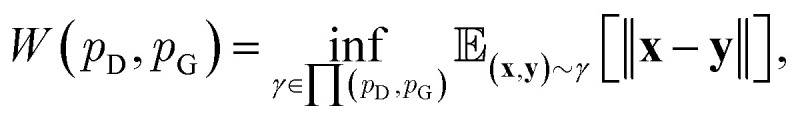
where 
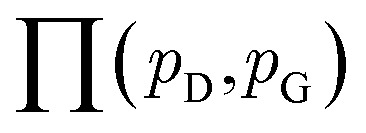
 is the set of all joint distributions *γ*(**x**,**y**) whose marginal distributions are 
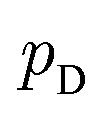
 and 
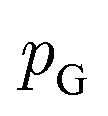
, respectively. Intuitively, Wasserstein distance measures the cost of transforming one distribution into another. This intractable distance 
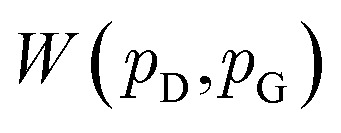
 can be approximated by:9
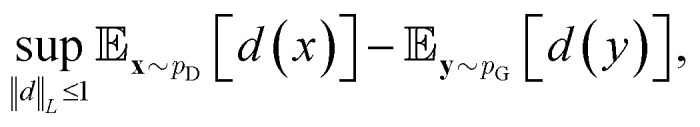
where 
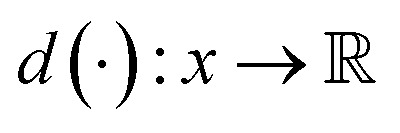
 belongs to 1-Lipschitz functions. In the context of WGANs, *d*(·) is the discriminator and we can write the objective of WGANs as:10
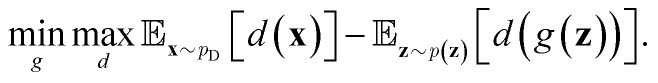


Note that the original discriminator of GANs is a binary classifier. On the other hand, in WGANs, the sigmoid function in the last layer of the discriminator should be removed in order to approximate the Wasserstein distance. In addition, the discriminator *d*(·) should satisfy the Lipschitz constraint. To this end, several approaches were proposed including weight clipping,^95^ gradient penalty,^96^ and spectral normalization.^97^ We refer the reader to these original papers for technical details. The smoother gradients provided by the loss function of WGANs can greatly improve the training process. Therefore, WGANs are widely used in various kinds of data generation tasks.^88,98,99^

Regarding peptide generation, several attempts have been made to utilize GANs and their variants to generate antimicrobial,^39,40,49,50^ anticancer,^48^ and immunogenic^51^ peptides. For example, upon training on 16K AMPs and 5K non-AMPs acquired from several datasets, PepGAN^50^ designed a mixed loss function to utilize AMP label information so that the discriminator was encouraged to distinguish real AMP sequences. Through experimental validation, the authors further showed that PepGAN was able to generate an AMP twice as strong as the conventional antibiotic ampicillin. In addition, AMPGAN^39^ and the follow-up model AMPGAN v2^40^ adopted a bidirectional CGAN framework to generate peptides with desired targets, mechanisms, and minimum inhibitory concentration (MIC) values. By introducing an encoder neural network to map peptide sequence to latent representations, the learnt latent space can be more structural and consequently leads to improved data modeling. Trained on around 6K–8K AMPs and 500K non-AMPs, AMP-GAN was able to generate three experimental validated AMPs. Its improved version, AMP-GAN v2, exhibited better training stability and generated a higher percentage of peptide antibiotic candidates, as predicted by several machine learning-based AMP classifiers. PandoraGAN^49^ utilized around 100–400 active antiviral peptides to generate novel peptides targeting viruses. To address the data scarcity problem, a novel training framework proposed in LeakGAN^100^ was adopted to provide richer signals and guide the model training during the intermediate process of peptide generation. Moreover, DeepImmuno-GAN^51^ used a WGAN with gradient penalty to generate immunogenic peptides that can bind to HLA-A*0201. GANDALF^48^ generated peptides that target cancer-related proteins such as PD-1, PDL-1, and CTLA-4. Specifically, GANDALF decomposed the peptide generation into sequence and structure generation steps and used two GANs to model these two generation processes. Despite the fact that GANs have been used to generate peptides with different properties, most still need wet-lab validation to support their effectiveness.

## Discussion

In this review, we summarize available deep generative models for peptides. Since peptide therapeutics have numerous applications, including in infectious diseases and cancer, the models described here represent foundational frameworks for the design of novel drugs. Recent advancements in software and hardware have allowed deep generative models to be trained at great speed and have enabled the generation of novel synthetic peptides displaying the desired properties. Such advances hold promise to accelerate peptide drug development by saving time, reducing cost, and increasing the likelihood of success. Indeed, the combination of generative models with deep neural networks has already generated promising peptides with therapeutic potential.

Despite these initial advances, several challenges still need to be addressed. For example, there is currently no single deep generative model framework that consistently yields better results compared to other deep generative models. As a result, selecting a suitable model from various deep generative frameworks can be difficult given a peptide dataset of interest. In addition, there is a lack of benchmarking datasets and metrics in peptide generation evaluation that further hinders model comparison and selection. In the area of molecular generation, benchmarking platforms like GuacaMol^101^ and MOSES^102^ have been developed to systematically evaluate the quality of generated data by using various metrics like novelty, uniqueness, validity, and Fréchet ChemNet Distance.^103^ There is an urgent need to establish similar platforms for benchmarking peptide generation models.

Apart from benchmarking platforms for generative model assessment, we discuss several future directions that can possibly lead to better peptide generation systems. (1) Better peptide property predictors/filters. To prioritize generated peptides for wet-lab validation, a number of generative methods require property predictors to filter and counterselect for undesired peptides. Consequently, the quality of property predictors can significantly influence the outcome of any peptide design project. Recent advances in multitask learning,^104^ transfer learning,^105^ and low-shot learning^106,107^ may be adopted to better utilize labeled peptide data and improve property predictors. In addition, by quantifying uncertainty of prediction (*e.g.*, peptide property predictions), active learning^108^ can select generated peptides with high uncertainty.^109^ In return, the experimental results of selected samples can be used as feedback to refine generative and property predictor models by efficiently expanding training data space and reducing model uncertainty. Similar to the case of generative model assessment, there is a lack of studies in ML-based peptide property prediction efforts aimed at systematically studying datasets, features, and model selection. Differences in dataset construction (*i.e.*, positive data selection and negative data generation) and a lack of comprehensive feature selection prevent direct comparison among various models.^110^ Similar to MoleculeNet^111^ in molecular ML, a peptide property prediction benchmark incorporating commonly used peptide datasets, peptide features (*e.g.*, physicochemical descriptors, one-hot encoding and learned representations) and ML models (*e.g.*, linear models, SVM, tree-based models and neural networks) can greatly help researchers to standardize the process of model evaluation and selection. (2) Further optimization on generated peptides. By combining deep generative models with optimization/searching methods like genetic algorithms, Bayesian optimization and *etc.*,^112–115^ generated samples can be further optimized to acquire improved properties and functions. Schissel *et al.*^57^ studied this notion to generate peptides using a deep generative model in combination with a genetic algorithm. (3) Incorporation of peptide structure information into deep generative models. While peptide structures can provide mechanistic information to better guide the model to generate peptides with desired functions, the majority of deep generative models (except Caceres-Delpiano *et al.*^54^ and Rossetto *et al.*^48^) in peptide design have only used sequence information. We hypothesize that the dynamic and flexible nature of peptide structures make them difficult to be inputted into deep generative models. For example, a single PDB structure of a peptide may be too static to capture sufficient information for computational modelling. Instead of using a single PDB structure, generative models that take a set of peptide structure conformers or a trajectory of structure changes (computed by molecular dynamics) as input may lead to more optimal peptide representation learning and subsequent peptide generation. With our increasing ability to generate peptide structural and functional data, coupled with advancements in deep learning, we anticipate that deep generative models will play a major role in drug discovery in years to come.

## Data availability

As this is a Review article, no primary research results, data, software or code have been included.

## Author contributions

Conceptualization: FW, DKH, CFN. Funding acquisition: CFN. Investigation: FW, DKH. Supervision: CFN. Visualization: FW. Writing – original draft: FW, DKH. Writing – review & editing: FW, DKH, CFN.

## Conflicts of interest

There are no conflicts to declare.

## Supplementary Material
